# A Probabilistic Bayesian Parallel Deep Learning Framework for Wind Turbine Bearing Fault Diagnosis

**DOI:** 10.3390/s22197644

**Published:** 2022-10-09

**Authors:** Liang Meng, Yuanhao Su, Xiaojia Kong, Xiaosheng Lan, Yunfeng Li, Tongle Xu, Jinying Ma

**Affiliations:** School of Mechanical Engineering, Shandong University of Technology, Zibo 255000, China

**Keywords:** wind turbine bearing, fault diagnosis, uncertainty, BayesianPDL

## Abstract

The technology of fault diagnosis helps improve the reliability of wind turbines. Difficulties in feature extraction and low confidence in diagnostic results are widespread in the process of deep learning-based fault diagnosis of wind turbine bearings. Therefore, a probabilistic Bayesian parallel deep learning (BayesianPDL) framework is proposed and then achieves fault classification. A parallel deep learning (PDL) framework is proposed to solve the problem of difficult feature extraction of bearing faults. Next, the weights and biases in the PDL framework are converted from deterministic values to probability distributions. In this way, an uncertainty-aware method is explored to achieve reliable machine fault diagnosis. Taking the fault signal of the gearbox output shaft bearing of a wind turbine generator in a wind farm as an example, the diagnostic accuracy of the proposed method can reach 99.14%, and the confidence in diagnostic results is higher than other comparison methods. Experimental results show that the BayesianPDL framework has unique advantages in the fault diagnosis of wind turbine bearings.

## 1. Introduction

Condition monitoring and fault diagnosis technologies for wind turbines have received more and more attention. According to the report of the Global Wind Energy Council, by the end of 2019, the total installed capacity of wind turbines has reached 651 GW [1]. Wind turbines have been under severe and extremely complex working conditions for a long time [2], resulting in a high component failure rate. Wind turbine bearing failures lead to long downtimes due to maintenance, and are prone to secondary failures, resulting in huge economic losses [3,4]. Therefore, fault diagnosis of bearings is of great significance for improving the operational productivity of wind turbines [5]. Currently, vibration signals are considered as an important source of information for monitoring the condition of bearings.

Huge amounts of data are generated during the operation of wind turbines [6]. How to mine effective fault information from massive data has become an urgent problem to be solved. Data-driven fault diagnosis models, e.g., multi-layer perceptron [7], rough set [8], Dempster–Shafer theory [9], support vector machine (SVM) [10], and deep learning [11], provide a new research direction for solving the above problems. Li et al. [12] proposed a local feature learning method based on backpropagation for rolling bearings fault diagnosis. Xu et al. [13] proposed an improved chaotic particle swarm optimization support vector machine method for fault diagnosis. The shallow learning machine is prone to overfitting during the training process, which leads to slow training speed and poor diagnostic effects. Deep learning provides a solution to solve the above problems. In order to solve the problems of insufficient extrapolation for the fault diagnosis of bearings in real wind turbines, a multi-scale convolutional neural network with bidirectional long short-term memory was designed by Xu et al. [14]. Kong et al. [15] proposed an enhanced sparse representation-based intelligent recognition method for planet bearing fault diagnosis in wind turbines. The proposed method makes the model highly interpretable. Xu et al. [16] proposed a fault diagnosis of the rolling bearing of wind turbines based on the variational mode decomposition and deep convolutional neural networks. A small sample fault diagnosis method for a wind turbine gearbox based on optimized generative adversarial networks was proposed by Su et al. [17]. Although the above method has achieved a good diagnostic effect, the output of the diagnostic result is the label of the class with the largest probability value, so it cannot give a certain degree of confidence for this classification result. Confidence in diagnostic results is challenged.

In manufacturing, we are interested in identifying any faulty machine accurately as early as possible. However, as with classification problems, traditional deep learning networks produce diagnostic outputs that are overconfident and unconfident. There are usually two kinds of mistakes, false negatives, and false positives. If the machine in normal operation is classified as a faulty machine, it is called a false negative. If the faulty machine is considered to be operating normally, it is called a false positive. In the first case, although production costs may be increased, the production can be resumed quickly. In the second case, huge losses in cost, time, and substandard products during production should be avoided. Fortunately, one can use the prediction uncertainty to fine-tune the decision of whether a machine is faulty or not. By quantifying the uncertainty, the confidence of the classification output is reflected.

Bayesian deep learning provides a novel way to make neural networks with uncertainty, giving diagnostic results with confidence [18]. Zhou et al. [19] applied the Bayesian deep learning to the fault diagnosis of bearings to achieve reliable diagnostic results, while verifying the transfer learning of neural networks. Maged et al. [20] exploited the prediction uncertainty information of Bayesian deep learning to improve fault detection. Tang et al. [21] used a Bayesian optimization algorithm to optimize the convolutional neural network and achieved certain results. Pérez-Pérez et al. [22] proposed a wind turbine uncertain model based on sensor data and an adaptive neuro-fuzzy inference system method. The above methods all use Bayesian deep learning to predict the uncertainty in the fault diagnosis process, but the traditional deep learning framework is not specially optimized. The traditional deep learning framework cannot identify the fault features of wind turbine bearings well, and there is a situation in which fault feature identification is lost during the identification process.

In summary, a probabilistic Bayesian parallel deep learning framework is proposed to achieve fault multiple classifications with high confidence. It solves the problems of difficulty in fault feature extraction and low confidence in diagnostic results of wind turbine bearing. To effectively extract the fault features of the wind turbine bearing, a parallel deep learning (PDL) framework is proposed. A Bayesian probability model is embedded in the PDL framework. The weights and biases in the PDL framework are then transformed from deterministic values to probability distributions to assess the confidence of the diagnostic results. The contributions of this paper are summarized as follows:

(1) The PDL framework is constructed to enhance the feature learning ability. Multiple parallel fusion residual blocks (PFRBs) are parallelized, which can enable the fault diagnosis performance. The PDL framework can adaptively select the number of PFRBs according to the characteristics of the dataset.
(2) A probabilistic Bayesian parallel deep learning framework fault diagnosis method is proposed under the framework of probabilistic Bayesian deep learning for the uncertainty perception of faults.
(3) Taking the fault signal of the gearbox output shaft bearing of a wind turbine in a wind farm as an example, it is proved that the proposed method has high accuracy and confidence in the diagnosis results. It exhibits excellent performance in the fault identification of wind turbine bearings.

The rest of this paper is as follows: Section 2 introduces the theoretical background and implementation of the Parallel deep learning framework. Section 3 details the overall implementation of the BayesianPDL framework. In Section 4, we conduct an experimental study on the fault signal of the gearbox output shaft bearing of a wind turbine in a wind farm, and compare the BayesianPDL framework with other methods in terms of fault diagnosis performance and confidence in the diagnosis results. Finally, the conclusions work of the research are discussed in Section 5.

## 2. Parallel Deep Learning Framework

When the wind turbine fault data are collected, due to the complex environment, the collected data signals contain a lot of redundant information and serious interference. Therefore, a parallel deep learning framework is proposed.

**Figure 1:**
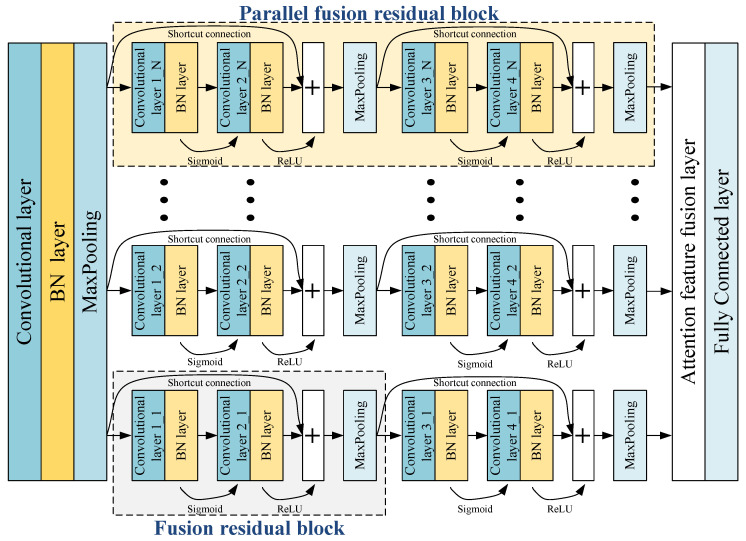
Parallel deep learning framework.

The PDL framework consists of multiple PFRBs: each PFRB consists of two fused residual blocks. Figure 1 shows the PDL framework. Feature maps are initially identified by a convolutional layer and pooling layer. The fault features information on the front layer is identified by PFRBs. The problem of network degradation is solved by shortcut connection. The fault features are fused in the attention feature fusion layer to achieve feature augmentation. The fault features are fitted in the fully connected layer to obtain fault diagnosis results.

### 2.1. Parallel Fusion Residual Block

**Figure 2:**
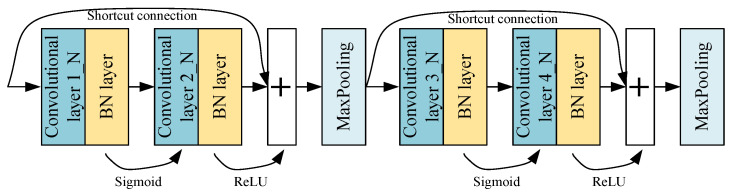
Parallel fusion residual block.

The fused residual block consists of two convolutional basic units and a max-pooling layer, and the convolutional basic unit is composed of a convolutional layer and a batch normalization layer. Different activation functions are used by different convolutional basic units. The PFRBs consist of two fused residual modules, as shown in Figure 2. Multiple PFRBs are parallelized and form the main PDL framework.

The output of a parallel fused residual block is:

(1) $$X_{1}^{PFRB}=X_{}^{\left(n,1\right)}+X_{}^{\left(n,2\right)},n=1,2,\ldots ,N$$

where $X_{1}^{PFRB}$ represents the output of a PFRB. $X^{\left(n,1\right)}$ and $X^{\left(n,2\right)}$ represent the output of the first fused residual block and the second fused residual block of the *n*th PFRBs, respectively. *N* is the number of PFRBs.

### 2.2. Attention Feature Fusion Layer

After the features are subjected to convolution, pooling, and other operations in PFRBs, the semantic information of the features can be effectively improved. It is necessary to distinguish fault features and noise before feature fusion. Focus on fault features and suppressing noise through an attention mechanism. The attention value of the fault feature is expressed as follows:

(2) $$A\left(\left(K,V\right),\varphi \right)=\sum_{i=1}^{\Delta }\frac{\exp\left(X_{i}^{T}\varphi /\sqrt{\delta }\right)}{\sum_{j}\exp\left(X_{i}^{T}\varphi /\sqrt{\delta }\right)}v_{i}$$

where ***A*** stands for attention value. $\left(K,V\right)=\left[\left(k_{1},v_{1}\right),\left(k_{2},v_{2}\right),\ldots ,\left(k_{\Delta },v_{\Delta }\right)\right]$ represents Δ th input information. ***K*** represents attention distribution, and ***V*** represents aggregated information. φ is the task query vector. δ represents the input dimension.

The high attention values of fault features are obtained under the action of the attention mechanism. High attention values have higher fusion weights, so feature augmentation is achieved when features are fused. Noise has a lower attention value. During feature fusion, the noise fusion weight is low, so it will not enhance.

The features extracted by each PFRBs are fused at the attention feature fusion layer according to different fusion weights. In this way, the feature augmentation of the internal network is realized. The output of the attention feature fusion layer is expressed as:

(3) $$X_{}^{PMBF}=Fusion\left(\left[\begin{array}{l} A^{\left(1,1\right)}X_{}^{\left(1,1\right)}+A^{\left(1,2\right)}X_{}^{\left(1,2\right)}\\ A^{\left(2,1\right)}X_{}^{\left(2,1\right)}+A^{\left(2,2\right)}X_{}^{\left(2,2\right)}\\ \begin{array}{cc} \ldots & \;\ldots \end{array}\\ A^{\left(n,1\right)}X_{}^{\left(n,1\right)}+A^{\left(n,2\right)}X_{}^{\left(n,2\right)} \end{array}\right],\xi \right),n=1,2,\ldots ,N$$

where $X_{}^{PMBF}$ is the output of the parallel fusion residual structure. ξ is the fusion weight.

## 3. Proposed Method

**Figure 3:**
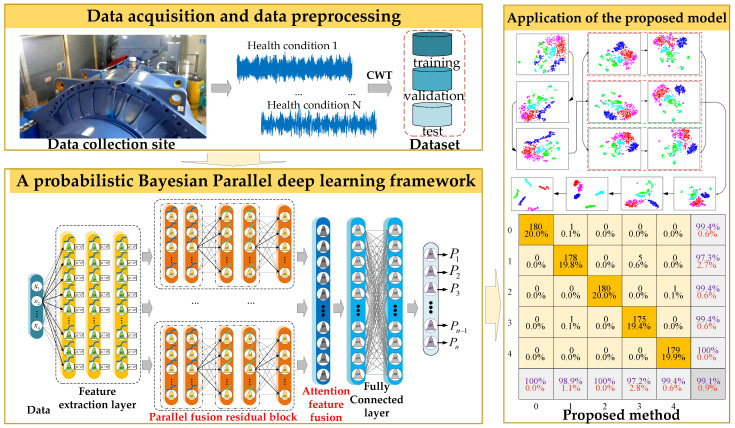
Fault diagnosis process of BayesianPDL framework.

In this study, an uncertainty-aware approach is explored in a probabilistic Bayesian deep learning framework for reliable machine fault diagnosis. Traditional deep learning has the problems of difficulty in fault feature extraction and low performance in fault diagnosis. Therefore, a probabilistic BayesianPDL framework is designed for the bearing fault diagnosis of wind turbines. The overall process of the proposed method is shown in Figure 3.

The general steps are summarized as follows:

Step 1: Signal acquisition by sensors. The raw signal is preprocessed using the CWT method. 

Step 2: According to the characteristics of the fault signal, select the appropriate number of PFRBs. This can eliminate noise interference components in the monitoring signal of wind turbines and capture more useful low-frequency fault features.

Step 3: BayesianPDL framework makes the posterior parameter distribution approximate to the variational distribution through variational inference, making the network uncertain.

Step 4: The training dataset trains the training network. The test dataset evaluates the confidence of the decisions given by the diagnostic model, resulting in a confident diagnosis.

### 3.1. A Probabilistic Bayesian Parallel Deep Learning Framework

**Figure 4:**
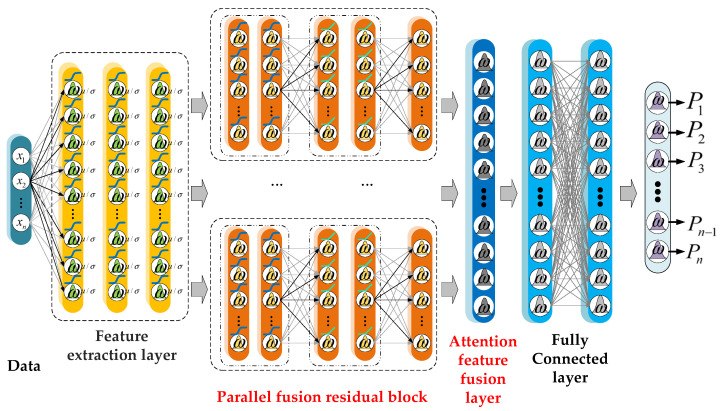
Bayesian parallel deep learning framework.

Figure 4 shows a probabilistic Bayesian parallel deep learning framework that uses weight distributions instead of point estimation in traditional deep learning. This gives it advantages for small dataset processing and network uncertainty. The model parameters during training, i.e., weights and biases, are parameterized to provide a probability distribution over all parameters, which is called the posterior distributions. The posterior distribution helps Bayesian neural networks capture uncertainty in network weights during classification [23]. It can make the prediction result have a higher confidence.

We define the model likelihood $p\left(y|f^{\omega }\right)$. Set dataset $X=\left\{x_{1},\;\ldots ,\;x_{N}\right\}$, $Y=\left\{y_{1},\;\ldots ,\;y_{N}\right\}$, [X,Y]∈D. To find the posterior distribution over parameters, the simple rules of probability, i.e., Bayes’ rule need to be used here:

(4) $$p(\omega |D)=\frac{p(D|\omega )p(\omega )}{p(D)}$$

where p(D|ω) represents the likelihood of data for a specific weight collection ω. p(D) is the marginal likelihood. p(ω) is the prior distribution.

### 3.2. Variational Inference

p(D) is difficult to calculate. Therefore, a simple distribution is used to approximate the posterior distribution, and the concept of approximation is introduced:

(5) $$p(\omega |D)\approx q_{\theta }(\omega |\theta )$$

The weights ω are sampled from a Gaussian distribution based on the parameter θ:$\omega ∼Ν(\mu_{\theta },\sigma_{\theta })$. 

Therefore, we need to find the minimum value between p(ω|D) and $q_{\theta }(\omega |D)$:

(6) $$\begin{array}{l} \theta^{opt}=\arg\underset{\theta }{\min}KL[q_{\theta }(\omega |\theta )||p(\omega |D)]\\ =\arg\underset{\theta }{\min}KL\left[q_{\theta }(\omega |\theta )||p(\omega )\right]-E_{q_{\theta }(\omega |\theta )}\left[\log\frac{q_{\theta }(\omega |\theta )}{p(D|\omega )p(\omega )}\right] \end{array}$$

where KL represents Kullback–Leibler divergence. $E_{q_{\theta }(\omega |\theta )}\left[\log p(D|\omega )\right]$ is likelihood cost. Find the minimum value of KL approximately equal to maximizing the evidence lower bound (ELBO), namely:

(7) $$L_{ELBO}(D,\theta )=E_{q_{\theta }(w|\theta )}\left[\log p(D|\omega )\right]-KL\left[q(\omega |\theta )||p(\omega )\right]$$

Approximate ELBO as an unbiased Monte Carlo estimator. Here, the tractable optimization object, i.e., maximization w.r.t.θ, becomes:

(8) $$L_{ELBO}(D,\theta )\approx \sum_{i=1}^{n}-\log p(D|\omega^{i})+\log q(\omega^{i}|\theta )-\log p(\omega^{i})$$

### 3.3. Uncertainty Analysis

Aleatoric uncertainty measures the amount of noise inherent in the data. Epistemic uncertainty in the weights can be reduced by observing more data [24]. This uncertainty induces prediction uncertainty by marginalizing over the (approximate) weights posterior distribution. 

To capture this uncertainty, we can sample multiple times from the distribution of model parameters to get T models, and use these T models to make predictions on the same sample. For a pixel *i* of the input features belonging to the category c, the model will output a prediction vector $f_{i}$. To avoid the integration of the weights, we approximate by two Monte Carlo sampling. This can be approximated using two Monte Carlo integrations as follows:

(9) $$\begin{array}{ll} p(y=c|x,\;X,\;Y) & \approx \frac{1}{T}\sum_{t=1}^{T}\frac{1}{1+\exp(-f^{\omega_{t}}(x))}\\ & \approx \frac{1}{T}\frac{1}{N}\sum_{t=1}^{T}\sum_{n=1}^{N}\frac{1}{1+\exp(-f^{\omega_{t}}(x))} \end{array}$$

where T samples masked model weights $\omega_{t}∼q_{\theta }^{}\left(\omega \right)$, $q_{\theta }^{}\left(\omega \right)$ is the Dropout distribution.

The uncertainty of this probability vector p can then be summarised using the entropy of the probability vector:

(10) $$\begin{array}{ll} H(p) & =-\sum_{c=1}^{C}p_{c}\log p_{c}\\ & \approx -\sum_{c=1}^{C}\frac{1}{T}\frac{1}{N}\sum_{t=1}^{T}\sum_{n=1}^{N}\frac{1}{1+\exp(-f^{\omega_{t}}(x))}\times \log\left(\frac{1}{T}\frac{1}{N}\sum_{t=1}^{T}\sum_{n=1}^{N}\frac{1}{1+\exp(-f^{\omega_{t}}(x))}\right) \end{array}$$

To sum up, the loss function of the training model is:

(11) $$L_{x}=\sum_{i}\log\frac{1}{T}\frac{1}{N}\sum_{t}\sum_{n}\exp\left(f_{i,c}^{\omega }+\sigma_{i}^{\omega }\varepsilon_{n,c}-\log\sum_{c^{\prime }}\exp\left(f_{i,c^{\prime }}^{\omega }+\sigma_{i}^{\omega }\varepsilon_{t,c^{\prime }}\right)\right)$$

where $f_{i}^{\omega }$ is the output when the network parameter is ω. In order for BayesianPDL framework to capture perceptual uncertainty, accidental uncertainty is captured by an additional addition of noise σ.

## 4. Case Studies and Results

### 4.1. Experimental Setup

**Figure 5:**
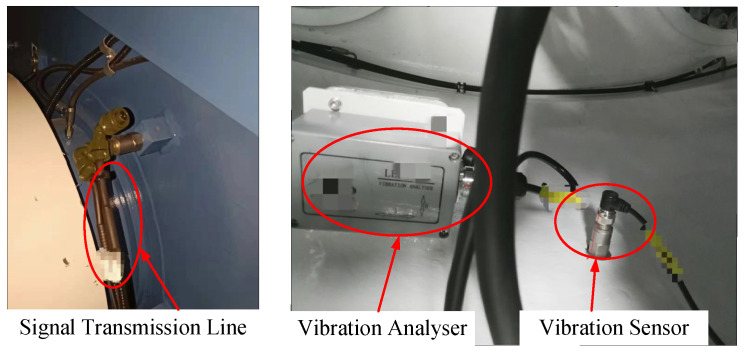
Signal acquisition.

In the experiment, the CTCWT135 vibration acceleration sensor was used to collect fault signals from the gearbox bearings of a wind turbine at a wind farm. This sensor is a dynamic range of ±10 g (peak), a sensitivity range of 500 (±10%) mV/g, and a measurement frequency responses range of 0.1 Hz to 10,000 Hz. The sampling frequency is set to 25,600 Hz, and the gearbox output shaft bearing type is NU2326. The sensor is fixed with a double-ended stud and installed on the gearbox wall directly above the bearing (12 o’clock). Figure 5 shows the vibration signal acquisition site.

**Figure 6:**
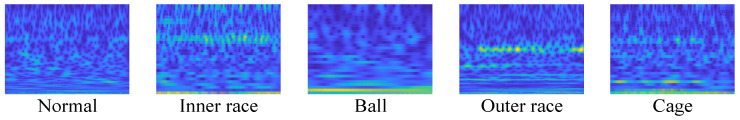
The time–frequency feature maps.

This experiment used five datasets and a total of five bearing states, including normal, inner race defect, ball defect, outer race defect, and cage defect. Among them, the inner ring failure is spalling, the failure of the ball and cage is worn, and the outer ring failure is electrical corrosion. Because the fault occurs at different times, the data are collected at different speeds. Detailed information on the dataset setting is shown in Table 1. As a time–frequency domain conversion method, CWT can effectively transform 1D vibration signals into 2D time–frequency maps which can be directly used by the convolution layers. Complex Morlet wavelet (cmor3-3), as a widely used WBF, is utilized in signal preprocessing. The bandwidth parameter and center frequency of the CWT are set to 3, and the length of the scale sequence is set to 128. The time–frequency feature maps are generated from the vibration signal, as shown in Figure 6.

The data samples are divided into a training dataset, validation dataset, and test dataset using the holdout cross-validation. The training dataset is used for the training model. The validation dataset is used to adjust and optimize parameters. The test dataset is used to evaluate the diagnostic performance of the BayesianPDL framework. To reduce the contingency of the experimental results, the results obtained are the average of five experimental results. 

The hyperparameters of the fused residual block are set as shown in Table 2, which consists of a convolutional layer, a batch normalization layer, and a max-pooling layer. For other parameter settings, the range of section depth is set to [1, 3], the range of initial learning rate is set to [1E-2, 1], the range of momentum is set to [0.8, 0.99], and the range of L2 regularization is set to [1E-10, 1E-2]. To make up for the lack of data volume, let the limited data generate more data, increase the number and diversity of training samples, and improve the robustness of the BayesianPDL framework. Commonly used methods include rotation reflection, flip, zoom, shift, scale, contrast, and so on. The rotation transformation method was selected in this experiment.

### 4.2. Experimental Results and Discussion

To obtain the best number of PFRBs, the proposed method can extract fault features from the noise background. The signal-to-noise ratio (SNR) is applied to represent the degree of noise added to the pure signal. The function of SNR is expressed as

(12) $$SNR=10\mathrm{lg}\left(\frac{P_{signal}}{P_{noise}}\right)\left(dB\right)$$

where $P_{signal}$ represents the power of the original signal. $P_{noise}$ represents the power of the added noise signal.

**Figure 7:**
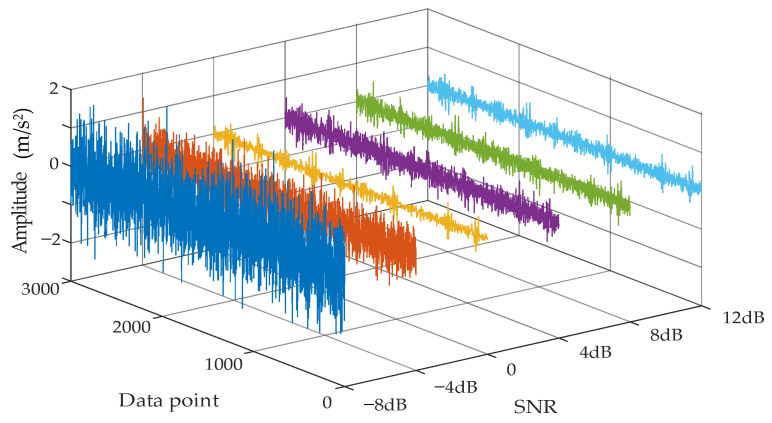
Comparison between different noisy vibration signals.

Figure 7 shows six different noisy signals. It can be found that different noisy signals have different amplitude characteristics. It can be seen from the figure that, as the SNR decreases, the vibration signal is obviously submerged in the noise.

**Figure 8:**
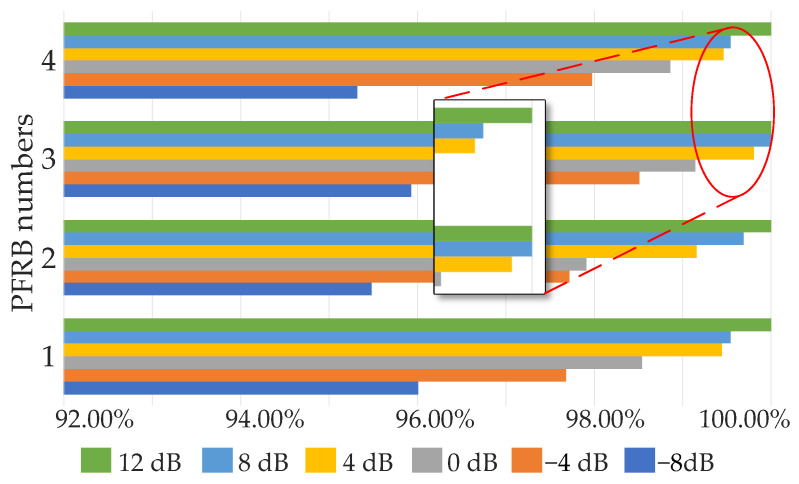
The accuracy of the BayesianPDL framework tested under different numbers of PFRBs.

The accuracy of the BayesianPDL framework tested under different numbers of PFRBs in different noise environments, as shown in Figure 8. The BayesianPDL frameworks with different numbers of PFRBs are trained using six kinds of noise signals. Each noisy signal is used to obtain the training dataset, the validation dataset and the testing dataset.

As the SNR gradually increases, the accuracy of the BayesianPDL framework is getting higher. As the number of PFRBs increase, the accuracy of the proposed method also increases. When the number of PFRBs is 3, the accuracy of fault diagnosis reaches the highest. 

**Figure 9:**
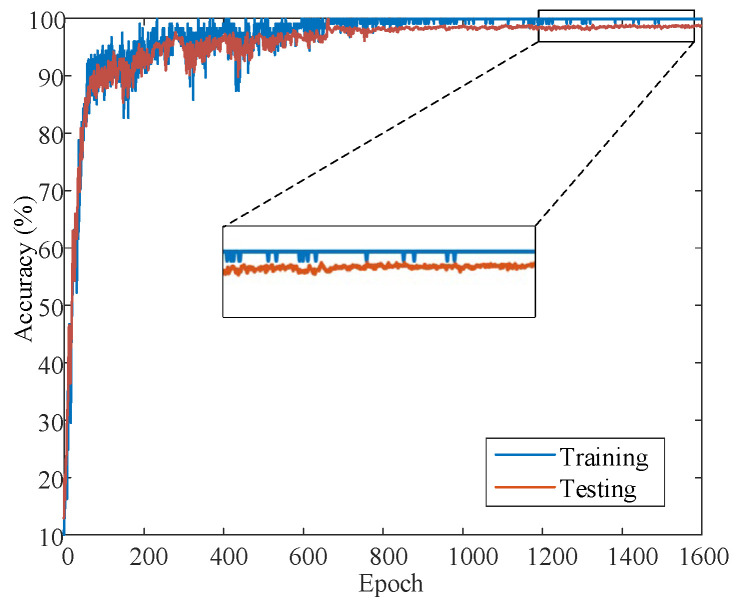
BayesianPDL framework training process.

Take SNR = 0 dB as an example, and observe its training curve. Figure 9 shows the convergence curve of the network training process when the number of PFRBs is 3. It can be intuitively found that the convergence curves of training and testing have a good fitting effect. The curve stops converging when the accuracy reaches 99.14%. From the testing curve, it is found that the proposed method is satisfactory. 

**Figure 10:**
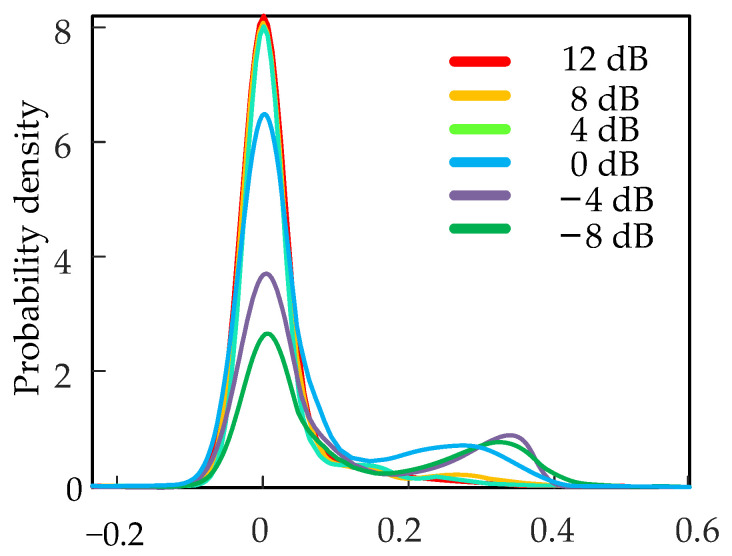
Total uncertainty distribution.

When PFRBs is 3, the uncertainty distribution of the network is shown in Figure 10. The probability density of the uncertainty distribution proposed method is significantly reduced due to the heavily disturbed noise on the signal at low SNR. This reduces the confidence of the diagnostic results and reduces the diagnostic accuracy. At higher SNR, the probability density is highly uniform with higher confidence. As SNR increases, so does the confidence of BayesianPDL, which gives high confidence in the diagnosis. Therefore, when the number of PFRBs is 3, the diagnostic results have the highest confidence and the highest accuracy, thus obtaining the optimal Bayesian PDL.

**Figure 11:**
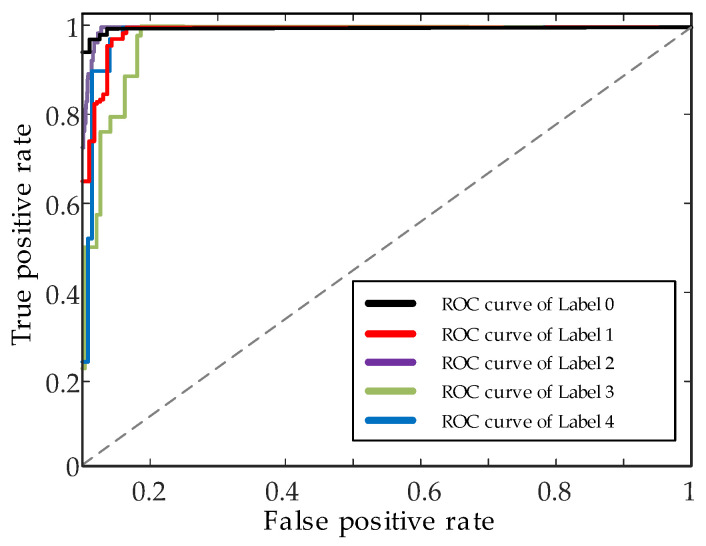
ROC curves for different labels.

**Figure 12:**
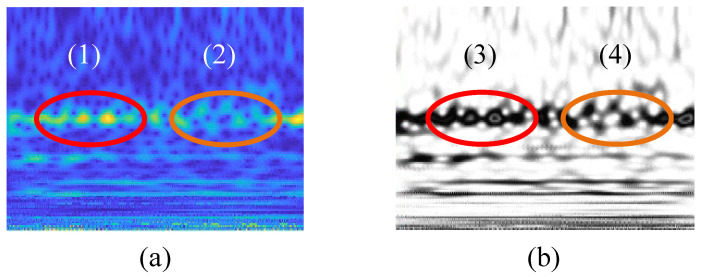
The outer ring fault features identified by BayesianPDL framework. (**a**) input feature map; (**b**) identified feature map. (1) and (2) is the fault feature of the input. (3) and (4) is the identified fault feature.

To further evaluate the predictive power of the model, receiver operating characteristic (ROC) curves of different fault labels are as shown in Figure 11. As a result, the BayesianPDL framework has a high fault detection capability. Figure 12 shows the outer ring fault features identified by the BayesianPDL framework. The input time–frequency feature map as shown in Figure 12a, and the features identified map by the BayesianPDL framework as shown in Figure 12b. The fault features of the outer ring are represented by feature (1) and feature (2), respectively, and the feature (2) is not obvious. However, the BayesianPDL framework can effectively identify fault features, such as feature (3) and feature (4), during the diagnostic process. This demonstrates that the proposed method has a strong feature extraction ability in the process of wind turbine bearing diagnosis.

In this experiment, EEMD, VMD, STFT, and ST are used to preprocess the raw signal, respectively. The preprocessed signal is used as the input to the optimal BayesianPDL framework. The CWT method significantly improved the feature resolution of the input BayesianPDL framework and achieved higher fault diagnosis accuracy. Table 3 shows the comparison results using other preprocessing methods as input to the optimal BayesianPDL framework. Among them, the input of the BayesianPDL framework is the raw one-dimensional vibration signal, and its fault diagnostic accuracy is the lowest. The CWT method significantly improved the feature resolution of the input BayesianPDL framework, and achieved higher fault diagnosis accuracy.

**Figure 13:**
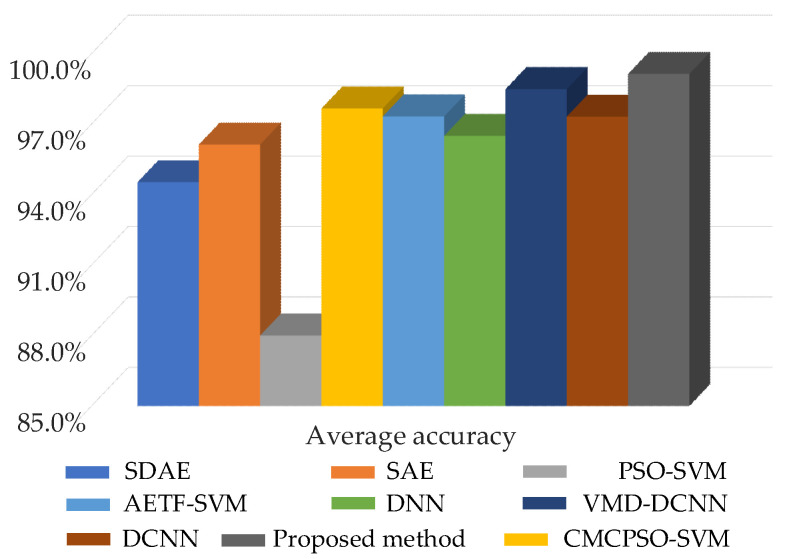
The testing diagnostic accuracy of different diagnostic methods.

Comparing the literature [13,25] and traditional machine learning fault diagnosis methods with optimal BayesianPDL, the input signal of different diagnostic methods is the raw vibration signal. Among them, the PSO-SVM method and the CMCPSO-SVM method were obtained through the literature 13. The AETF-SVM method was obtained through the literature 25. Figure 13 shows the average diagnostic accuracy of different diagnostic methods. Compared with other diagnostic methods, the method proposed has the highest accuracy. However, the other methods are all non-probabilistic diagnostic models (the network is deterministic). When the diagnostic process with the confidence level cannot be obtained, this results in unreliable diagnostic results.

**Figure 14:**
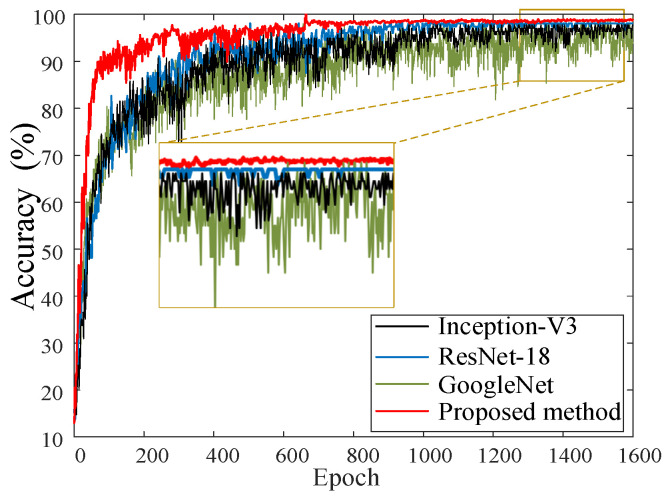
The accuracy convergence curves of different diagnostic methods.

In the Bayesian deep learning framework, the Bayesian process can be flexibly transferred to other types of deep learning models. Compare the convergence performance of the optimal BayesianPDL framework with GoogleNet, ResNet-18, and Inception-V3 methods. The accuracy convergence curves of the raw signal input for different diagnostic methods are shown in Figure 14. It can be seen from the four convergence curves that the proposed method has the fastest convergence speed, the smallest curve fluctuation, and the best convergence effect. There are many fluctuations in the other three methods during the training process, which indicates that there may be problems such as gradient explosion during network fitting. The convergence curve of GoogleNet still has large fluctuations after the diagnosis, resulting in a diagnostic result of only 97.21%.

**Figure 15:**
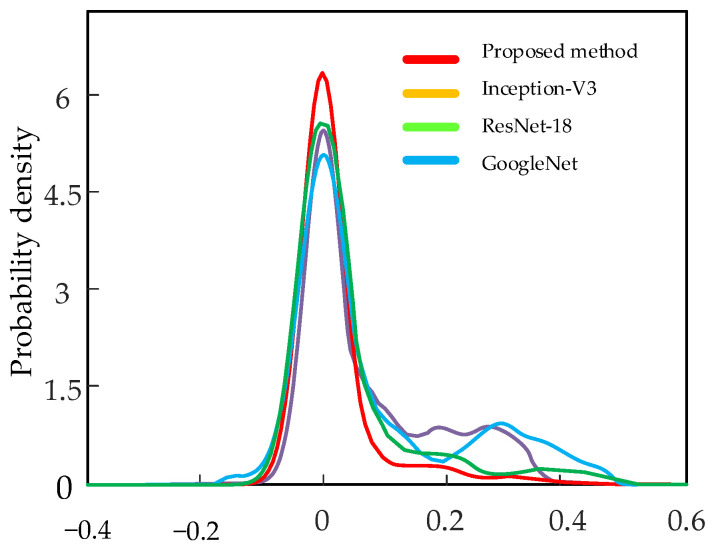
Uncertainty distribution for different methods.

**Figure 16:**
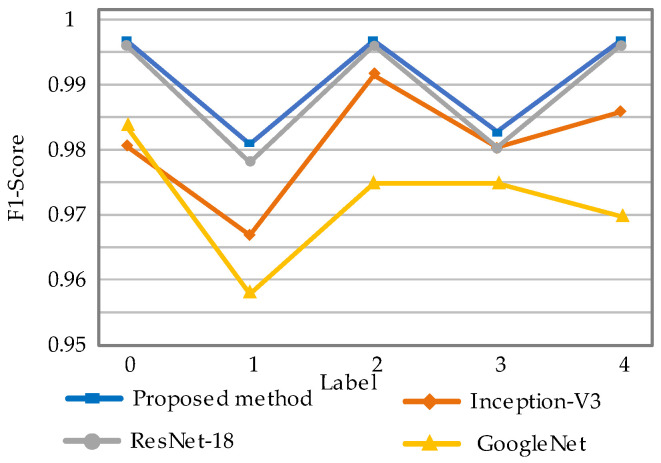
F1-score for different labels.

**Figure 17:**
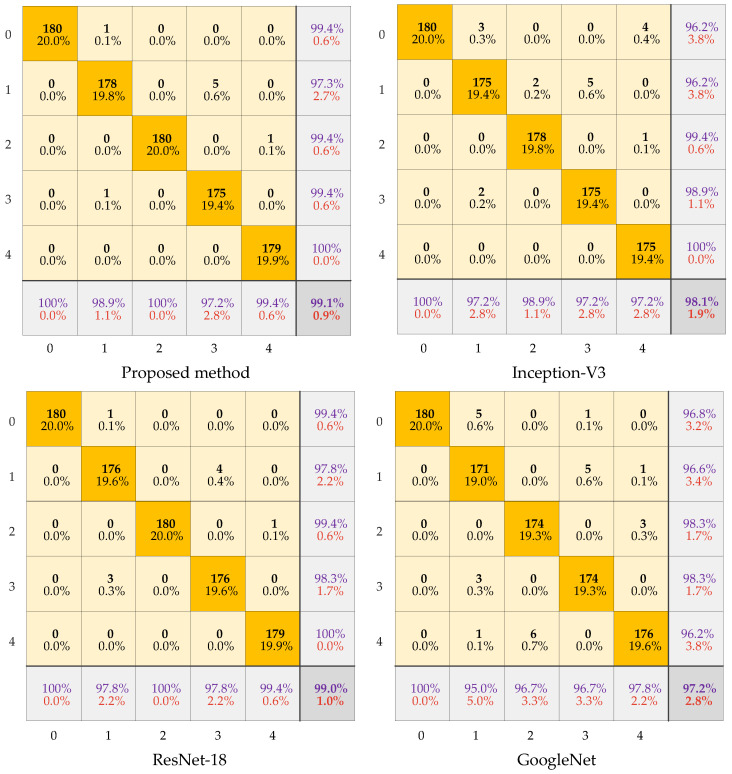
Confusion matrix for different methods.

The uncertainty contributions of different diagnostic models are as shown in Figure 15. The proposed method has a higher probability density. This indicates higher confidence in the diagnostic results of the proposed method. The F1-score of the different fault labels is represented in Figure 16. It can be seen that the method proposed has the highest F1-score and thus the best quality of the model. The confusion matrix of the four models is shown in Figure 17. For each type of label, the method proposed has high diagnostic accuracy. Therefore, the BayesianPDL framework has superior diagnostic capability to other signal analysis methods.

The training time of the optimal BayesianPDL framework is compared with that of GoogleNet, ResNet-18, and Inception-V3, methods. The efficiency of raw vibration signal input into different diagnostic methods is shown in Table 4. As the number of PFRBs increases, the training time of the BayesianPDL framework continues to increase. However, the accuracy is highest when the BayesianPDL framework has three PFRBs. Other diagnostic methods have a long training time due to the complex network structure. The proposed method has high accuracy and the shortest training time.

T-SNE (T-distributed stochastic neighbor embedding) is a nonlinear dimensionality reduction algorithm, which can map the data in the high-dimensional space to the low-dimensional space and retain the local characteristics of the data set. For the visualized data, the spacing between similar data sets is small and the spacing between heterogeneous data sets is large.

**Figure 18:**
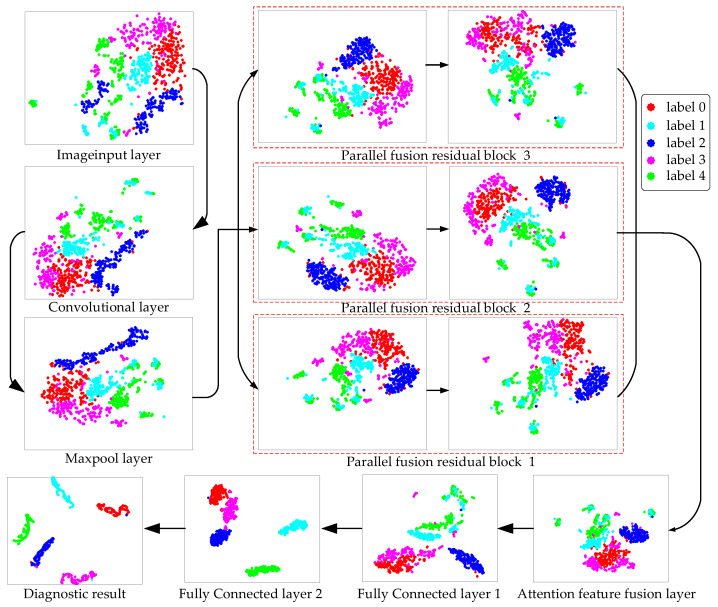
T-SNE visualization of bearings.

High-dimensional features are mapped to two-dimensional space by the T-SNE algorithm, as shown in Figure 18. The changes in data points within the optimal BayesianPDL framework are visually displayed by the T-SNE algorithm. As data points are entered, they cross each other and are distributed chaotically. With the continuous deepening of feature extraction, data points in PFRBs begin to be fitted and classified, and some features are separated. In the attention feature fusion layer, the data points are fused according to different attention values to achieve feature augmentation inside the network. The fault points are classified at the fully connected layer. All types of faults are classified clearly and the diagnostic results are obtained.

## 5. Conclusions

Fault diagnosis of wind turbines plays an important role in improving the reliability of wind turbines. To address the problems of difficult feature extraction and low confidence of diagnostic results in traditional deep learning for wind turbine bearing faults, a probabilistic Bayesian parallel deep learning framework fault diagnosis method is proposed. Instead of implementing manual feature extraction and selection, the proposed method directly uses raw vibration signals to carry out fault diagnosis in an end-to-end way, greatly reducing the reliance on human expertise and manual intervention. In a nutshell, the advantages of the proposed wind turbine fault diagnosis that should be highlighted are:

(1) A structure of PFRBs is constructed to enrich high-level feature data. The fault feature extraction ability of the PDL framework is improved without increasing the network parameters. Through the attention mechanism, useful information is identified by the network in the extracted features.
(2) Based on the PDL framework, the hyperparameters of the network are parameterized, providing the probability distribution of all hyperparameters, which makes the neural network uncertain. A probabilistic BayesianPDL framework diagnostic method specially applied to wind turbines bearing faults is designed.
(3) Compared with other non-probabilistic models, the proposed method has a higher diagnostic performance. Compared with other probability models, the diagnostic results of the proposed method have higher accuracy and confidence.

## Figures and Tables

**Table 1:** Rolling bearing fault dataset.

Labels	Fault Location	Rotating Speed	Dateset (Training/Validation/Test)
0	Normal	1482	600/200/180
1	Inner race	1717	600/200/180
2	Ball	1326	600/200/180
3	Outer race	1788	600/200/180
4	Cage	1831	600/200/180

**Table 2:** Fusion residual block hyperparameters settings.

No. of Layer	Layer	Parameters
Conv1_1	Convolution layer 1_1	64 convolution kernels with the size of [3,3]. Stride: [2,2]. Padding: same.
BN	Batch Normalization layer	Scale is 64.
Conv2_1	Convolution layer 2_1	64 convolution kernels with the size of [3,3]. Stride [2,2]. Padding: same
BN	Batch Normalization layer	Scale: 64.
MaxPooling	MaxPooling layer	Pooling size: [3,3]. Stride: [2,2]. Padding: [0,0,0,0].

**Table 3:** Test results using different data preprocessing methods.

Methods	Testing Accuracy
BayesianPDL	96.54% ± 0.3168
EEMD-BayesianPDL	97.93% ± 0.1799
VMD-BayesianPDL	98.01% ± 0.1997
STFT-BayesianPDL	97.56% ± 0.3752
ST-BayesianPDL	97.48% ± 0.2688
Proposed method	99.14% ± 0.0401

**Table 4:** The diagnostic performance of the different methods.

Methods	Testing Accuracy	Training Time (s)
Proposed method (one PFRB)	98.15% ± 0.1136	1954
Proposed method (two PFRB)	98.91% ± 0.1039	3018
Proposed method (three PFRB)	99.14% ± 0.0401	3780
GoogleNet	97.21% ± 0.0719	5982
ResNet-18	99.08% ± 0.0724	5540
Inception-V3	98.12% ± 0.0439	6078

## Data Availability

The data presented in this work are available on request from the corresponding author.
